# Supplementary material for: WNT-induced association of Frizzled and LRP6 is not sufficient for the initiation of WNT/β-catenin signaling
Source: Nat Commun. 2025 May 24;16:4848. doi: 10.1038/s41467-025-60096-7 (PMC12103576; doi:10.1038/s41467-025-60096-7)
Supplement: Supplementary file 1 — Supplementary Information [file 41467_2025_60096_MOESM1_ESM.pdf]

Supporting information for:

WNT-induced association of Frizzled and LRP6 is not sufficient for the initiation of WNT/β-catenin signaling

Jan Hendrik Voss[1], Zsombor Koszegi[2], Yining Yan[1], Emily Shorter[3], Lukas Grätz[1], Johanna T. Lanner[3], Davide Calebiro[2], Gunnar Schulte[1*]

Affiliations:

[1]Karolinska Institutet, Department of Physiology & Pharmacology, Sec. Receptor Biology & Signaling, Biomedicum, S-17165 Stockholm, Sweden

[2]Department of Metabolism and Systems Science, College of Medicine and Health, University of Birmingham, Birmingham B15 2TT, UK

[3]Centre of Membrane Proteins and Receptors (COMPARE), Universities of Nottingham and Birmingham, Birmingham B15 2TT, UK

[4]Karolinska Institutet, Department of Physiology & Pharmacology, Sec. Molecular Muscle Physiology & Pathophysiology, Biomedicum, S-17165 Stockholm, Sweden

[*] Corresponding author; gunnar.schulte@ki.se

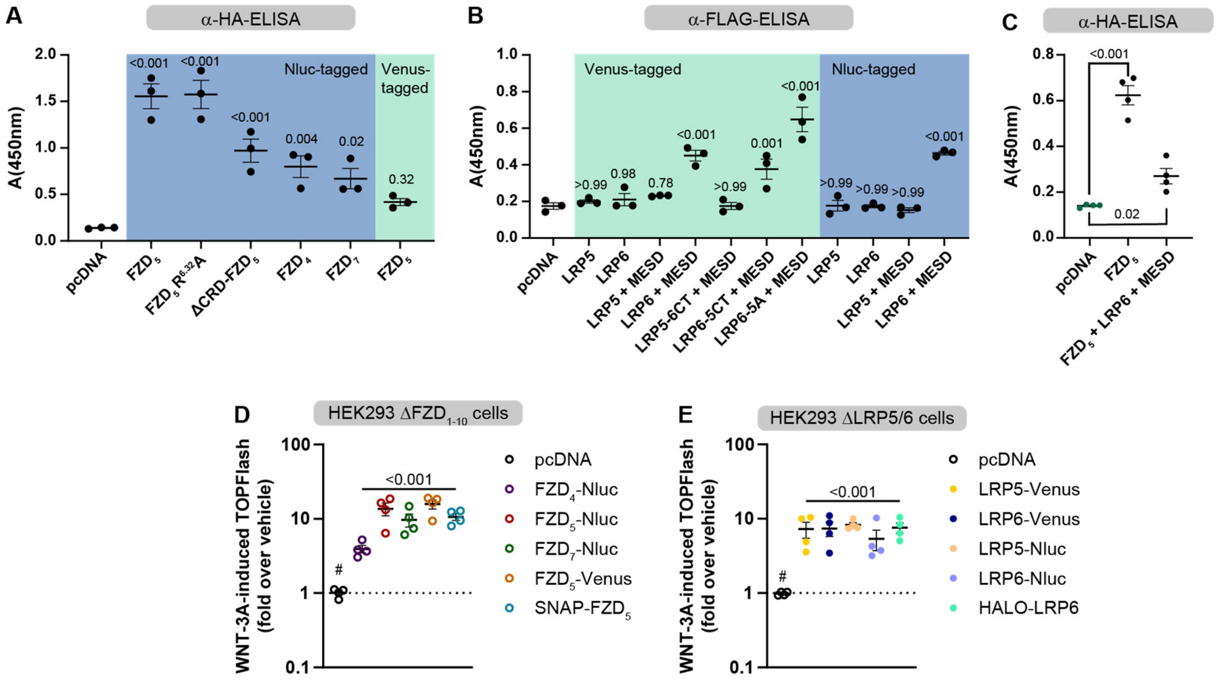

Supplementary Figure 1. Validation of constructs employed in the study. Surface expression in HEK293 cells was determined by surface ELISA employing the N-terminal HA-tags (for FZDs) or FLAG-tags (for LRPs). **A.** Surface expression of HA-FZD-Nluc/Venus constructs. **B.** Surface expression of FLAG-LRP-Nluc/Venus constructs. **C.** Co-expression of LRP6-Venus reduces FZD_5-Nluc surface expression, which stays in a detectable range. **D, E.** TOPFlash reporter gene assay of HEK293 $\Delta FZD_{1\text{-}10}$ cells transfected with tagged FZD constructs (D) and HEK293 ΔLRP5/6 cells transfected with tagged LRP constructs (E) used throughout this study. Cells were stimulated with 300 ng/ml WNT-3A for 24 h before measurement. Data are presented as a mean ± SEM of three (A, B) or four to five (C, D, E) independent experiments, each performed in technical triplicates. Statistical significance was assessed via a one-way ANOVA with Dunnett's post-hoc test with *p*-values indicated in the figure (for C, a matched ANOVA was employed; for reporter gene assays (D,E), data were log10-transformed prior to analysis).

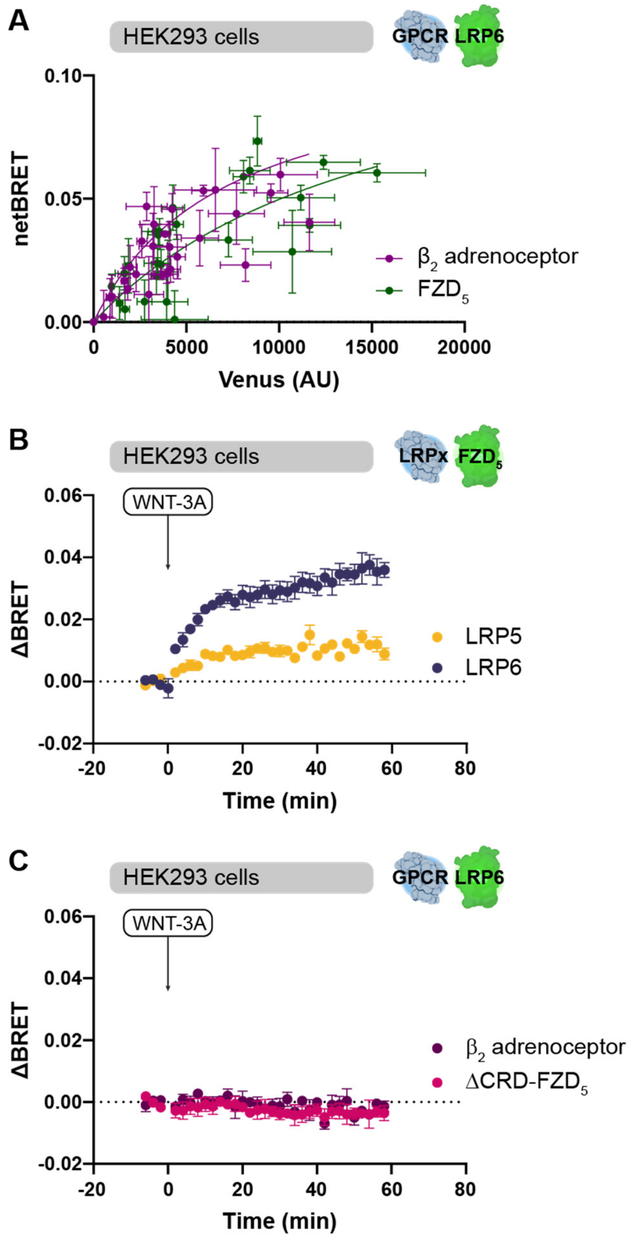

Supplementary Figure 2. FZD$_5$ and LRP6 form a dynamic, but not a constitutive complex. **A.** Acceptor titration experiments with LRP6-Venus using β$_2$-adrenoceptor-Nluc (magenta) and FZD$_5$-Nluc (green) as donors. **B.** Kinetic BRET experiments performed with an inverse probe setup (LRP5/6-Nluc (yellow/blue) and FZD$_5$-Venus). **C.** Absence of dynamic interaction between β$_2$-adrenoceptor-Nluc (purple) and ΔCRD-FZD$_5$-Nluc (magenta) with LRP6-Venus upon WNT-3A stimulation (1000 ng/ml). Data points are merged from three independent experiments (A; error bars represent the SD of three technical replicates), or shown as a mean ± SEM of three independent experiments each performed in triplicate (B, C).

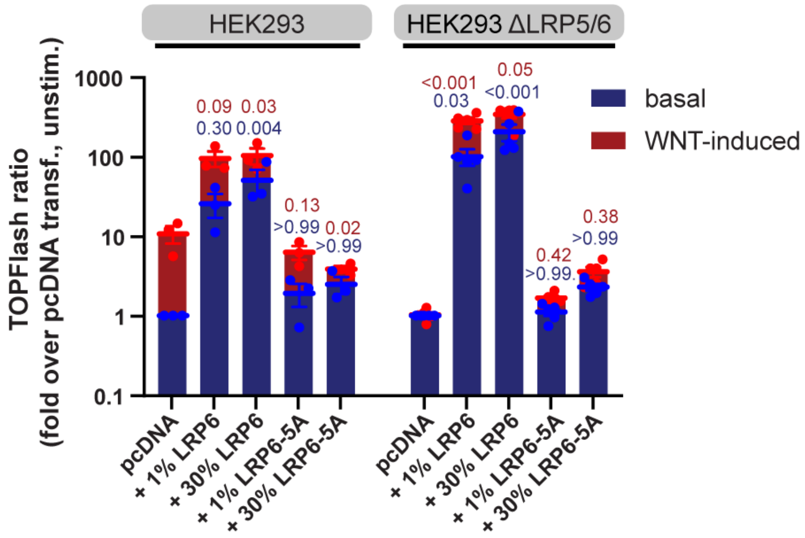

Supplementary Figure 3. The LRP6-5A phosphorylation-deficient mutant is functionally inactive. TOPFlash reporter gene experiments showing basal (blue) and WNT-3A-induced (red) TOPFlash reporter gene expression in HEK293 or HEK293 ΔLRP5/6 cells transfected as indicated. Data were normalized to the respective pcDNA-transfected, unstimulated condition of each cell line for each biological replicate. Bar graphs display mean ± SEM of four independent experiments performed in triplicate. Statistical comparisons were made by a one-way ANOVA followed by Dunnett's post-hoc test, compared to each group's pcDNA-transfected sample. In the untreated samples (blue), the pcDNA-transfected sample of each group served as a reference for the multiple comparisons. For the WNT-induced samples (red), the fold-increase of the WNT-3A stimulated condition over the basal condition was subjected to statistical analysis.

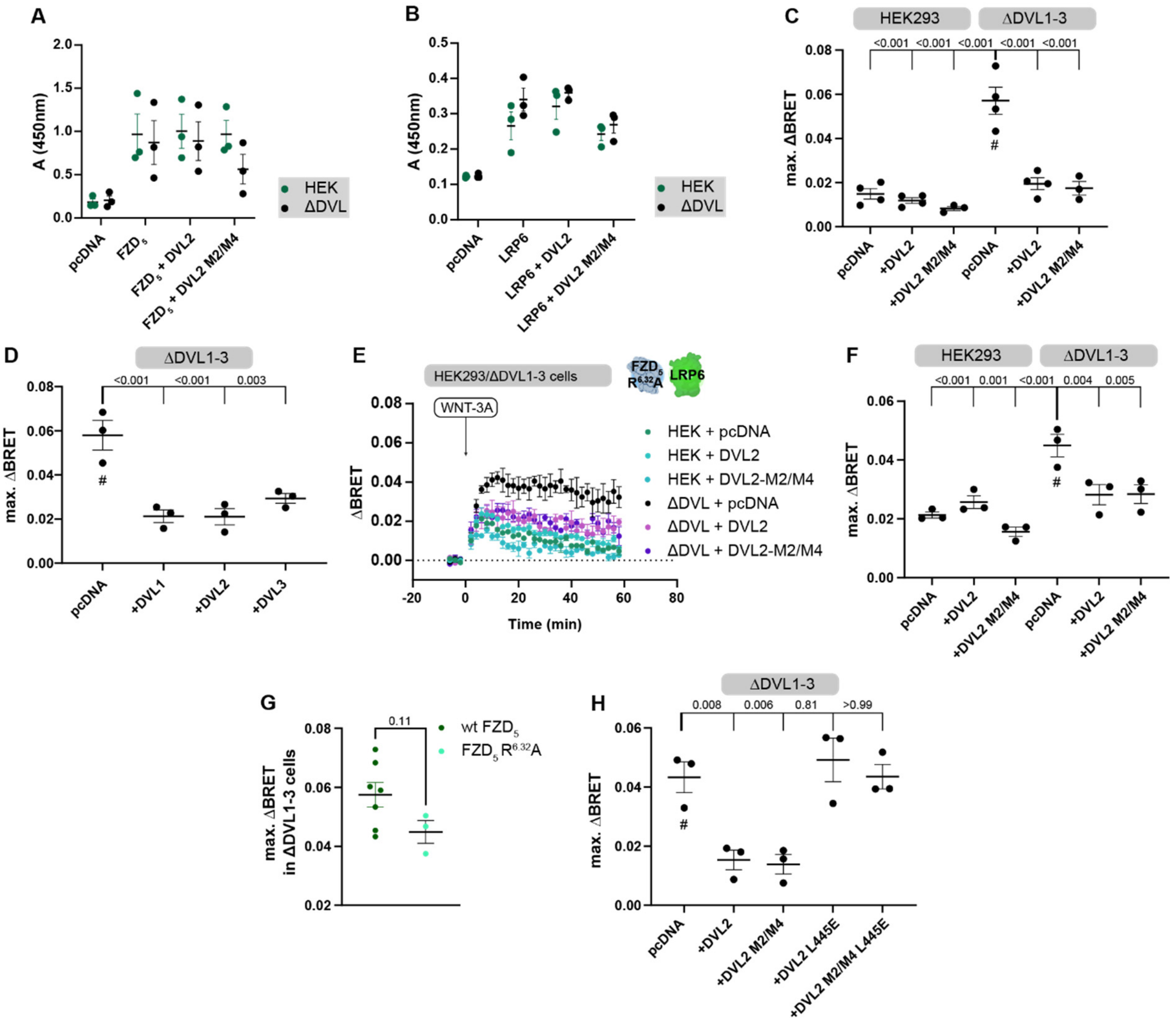

Supplementary Figure 4. DVL-FZD interactions reduce the dynamic ΔBRET range between FZD$_5$ and LRP6 upon WNT-3A stimulation. **A**. Surface expression of FZD$_5$-Nluc is not decreased upon co-expression of DVL2 or DVL2 M2/M4 in HEK293 (dark teal) and ΔDVL1-3 cells (black) as determined by anti-HA surface ELISA. **B**. Surface expression of LRP6-Venus is not decreased upon co-expression of DVL2 or DVL2 M2/M4 in HEK293 and ΔDVL1-3 cells as determined by anti-FLAG surface ELISA. **C**. ΔBRET$_{max}$ in HEK293 cells and ΔDVL1-3 cells transfected either with the BRET sensor pair alone or additionally with DVL2 or DVL2 M2/M4. Paired with Fig. 2C. **D**. ΔBRET$_{max}$ values of ΔDVL1-3 cells transfected either with the FZD$_5$-LRP6 BRET sensor pair and pcDNA, DVL1, DVL2, and DVL3 stimulated with 1000 ng/ml WNT-3A. Paired with Fig. 2D. **E**. Kinetic ΔBRET traces of HEK293 or ΔDVL1-3 cells transfected with FZD$_5$ R$^{6.32}$A-Nluc, LRP6-Venus, and either pcDNA, DV L2, or DVL2-M2/M4 as indicated, stimulated with 1000 ng/ml WNT-3A. **F**. ΔBRET$_{max}$ values of HEK293 or ΔDVL1-3 cells transfected with the BRET sensor pair and pcDNA, DVL2, or DVL2 M2/M4. Paired with Supp. Fig. 3D. **G**. The observed WNT-3A-induced ΔBRETmax is not significantly different in ΔDVL1-3 cells if a FZD$_5$ R$^{6.32}$A-Nluc donor is used compared to wt FZD$_5$-Nluc. ΔBRETmax values taken from Supp. Fig. 4C/D (wt FZD$_5$; n=7) and Supp. Fig. 4E (FZD$_5$ R$^{6.32}$A; n =3) and Fig. 2C. Significance was assessed by a two-sided unpaired t-test; not significant = $p > 0.05$. **H**. ΔBRET$_{max}$ values of ΔDVL1-3 cells

transfected either with the BRET sensor pair and pcDNA, DVL2, DVL2 M2/M4, or the L445E mutants of these DVL2 constructs. Paired with Fig. 2E. Statistical analyses were performed by one-way ANOVA and Dunnett's post-hoc test, except for panel G, where a two-tailed unpaired *t*-test was employed. # indicates the group used as reference for multiple comparisons. *p*-values are provided in the figure. Data are shown as mean ± SEM of three (A, B, D, E, F, H) or four (C) independent experiments performed in triplicate.

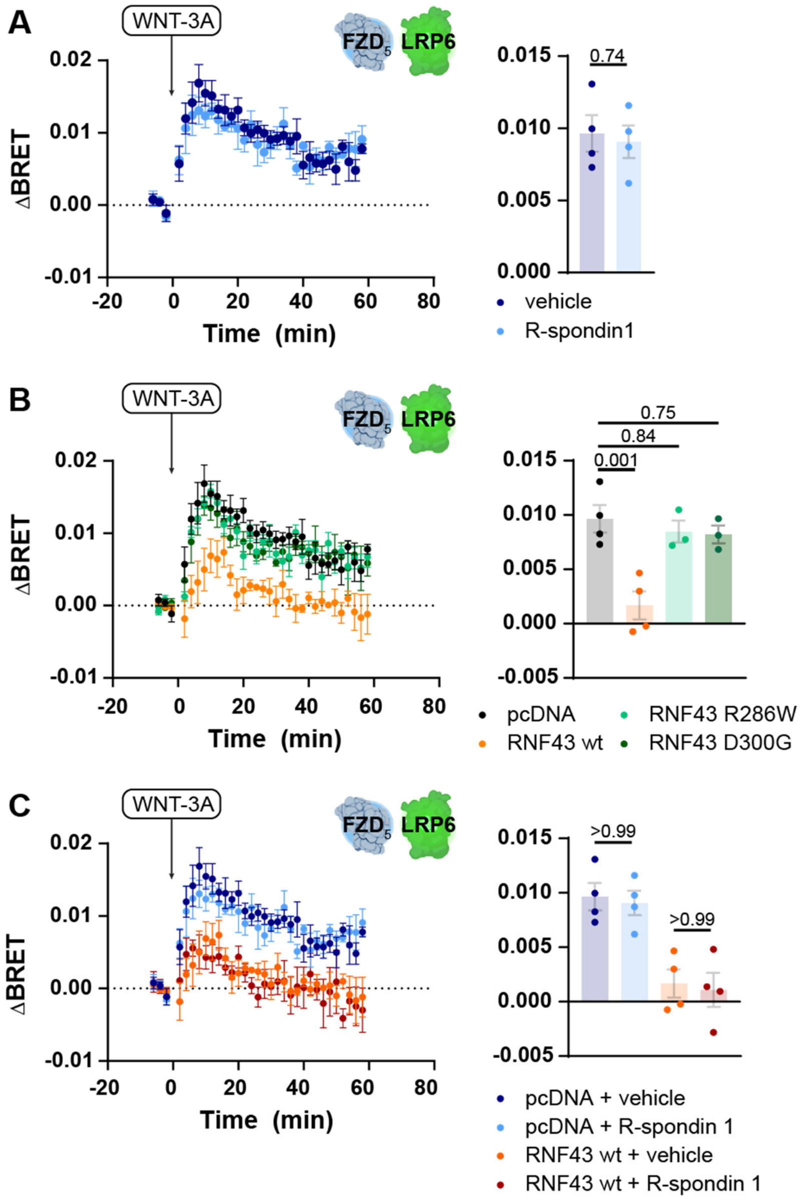

Supplementary Figure 5. RNF43 co-expression blunts BRET assessment of FZD_5-LRP6 association independent of R-spondin 1 treatment. A. Kinetic ΔBRET traces and average ΔBRET values of HEK293 cells transfected with FZD_5-Nluc and LRP6-Venus. Cells were pre-incubated for 2 h with either vehicle or 100 ng/ml R-spondin 1, and subsequently stimulated with 1000 ng/ml WNT-3A. **B.** Kinetic ΔBRET traces and average ΔBRET values of HEK293 cells transfected with FZD_5-Nluc, LRP6-Venus, and RNF43 (wt and the dominant-negative R286W and D300G mutants), stimulated with 1000 ng/ml WNT-3A. **C.** Kinetic ΔBRET traces and average ΔBRET values of HEK293 cells transfected with FZD_5-Nluc, LRP6-Venus, and either pcDNA or wt RNF43. Cells were pre-incubated for 2 h with either vehicle or 100 ng/ml R-spondin 1 and subsequently stimulated with 1000 ng/ml WNT-3A. Data are shown as mean ± SEM of three or four independent experiments (see on the bar graph on the right side), each performed in triplicate. Average ΔBRET values were analyzed with either an unpaired two-tailed *t*-test (A) or a one-way ANOVA followed by Sidak's multiple comparisons, where the mean of each group is compared with the mean of every other group (B, C). *p*-values are displayed in the figure for comparisons of interest.

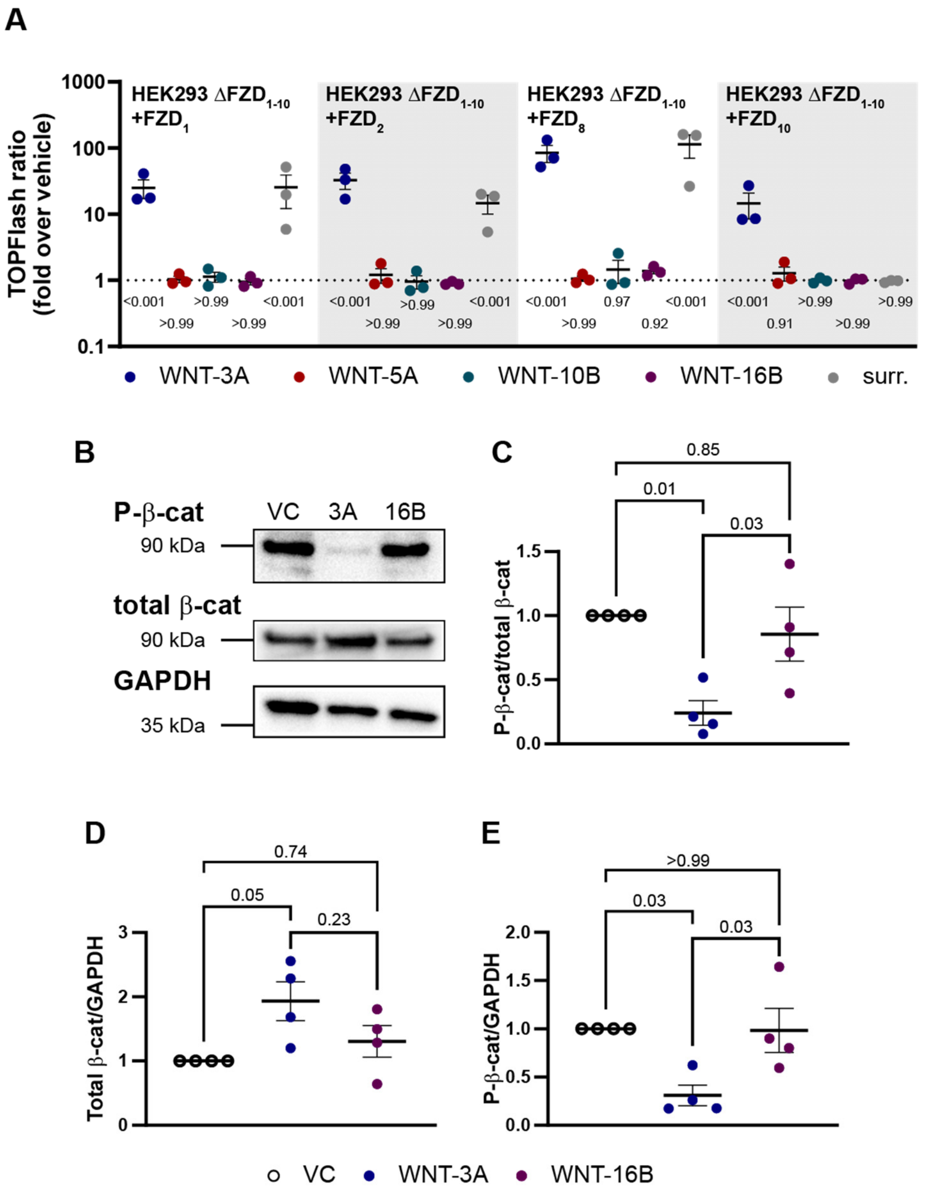

Supplementary Figure 6. WNT-16B does not activate the TOPFlash reporter gene assay and does not stabilize β-catenin. A. TOPFlash reporter gene assays performed in HEK293T ΔFZD$_{1-10}$ cells overexpressing FZD$_{1/2/8/10}$, stimulated with diverse WNTs (300 ng/ml) and WNT surrogate (1 nM) for 24 h. **B.** Immunoblotting of phospho-β-catenin, total β-catenin and GAPDH (loading control) from whole cell lysates of HEK293 cells stimulated for 2 h with 300 ng/ml WNT-3A, WNT-16B or a vehicle control. **C-E.** Densitometric analyses of the ratios of phospho-β-catenin to total β-catenin (C), total β-catenin to GAPDH (D), and phospho-β-catenin to GAPDH (E) show that WNT-3A, but not WNT-16B stabilizes β-catenin as evidenced by a reduction in phosphorylated β-catenin and elevation of total β-catenin levels. *P*-values for reporter gene assay data were obtained by a one-way ANOVA of log10-transformed data followed by Dunnett's post-hoc test (A). *P*-values for densitometric analyses were obtained with a one-way ANOVA followed by Sidak's multiple comparisons test comparing all samples with each other (C-E).

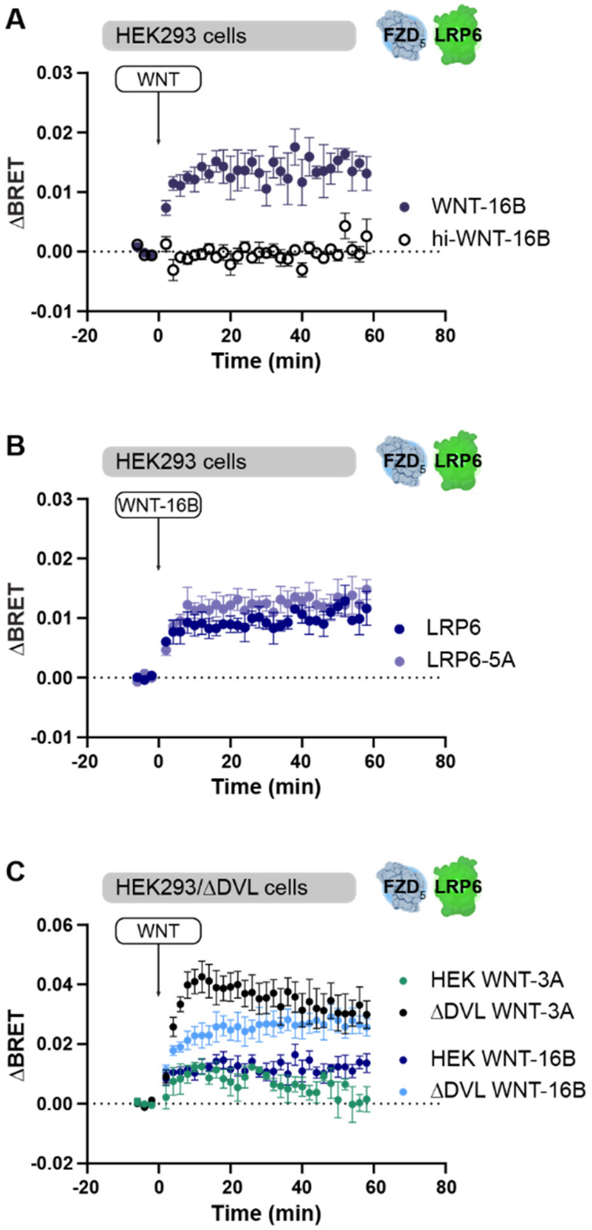

Supplementary Figure 7. WNT-16B behaves similar to WNT-3A in FZD_5-LRP6-BRET assays. **A**. Kinetic ΔBRET traces of 1000 ng/ml WNT-16B or heat-inactivated WNT-16B (hi-WNT-16B) in HEK293 cells transfected with FZD_5-Nluc and LRP6-Venus. **B**. Kinetic ΔBRET traces of 1000 ng/ml WNT-16B in HEK293 cells transfected with FZD_5-Nluc and LRP6- or LRP6-5A-Venus. **C**. Direct comparison of kinetic ΔBRET traces of HEK293 and ΔDVL cells transfected with FZD_5-Nluc and LRP6-Venus stimulated with 1000 ng/ml WNT-3A or WNT-16B. All data points are shown as mean ± SEM of three individual experiments performed in triplicate.

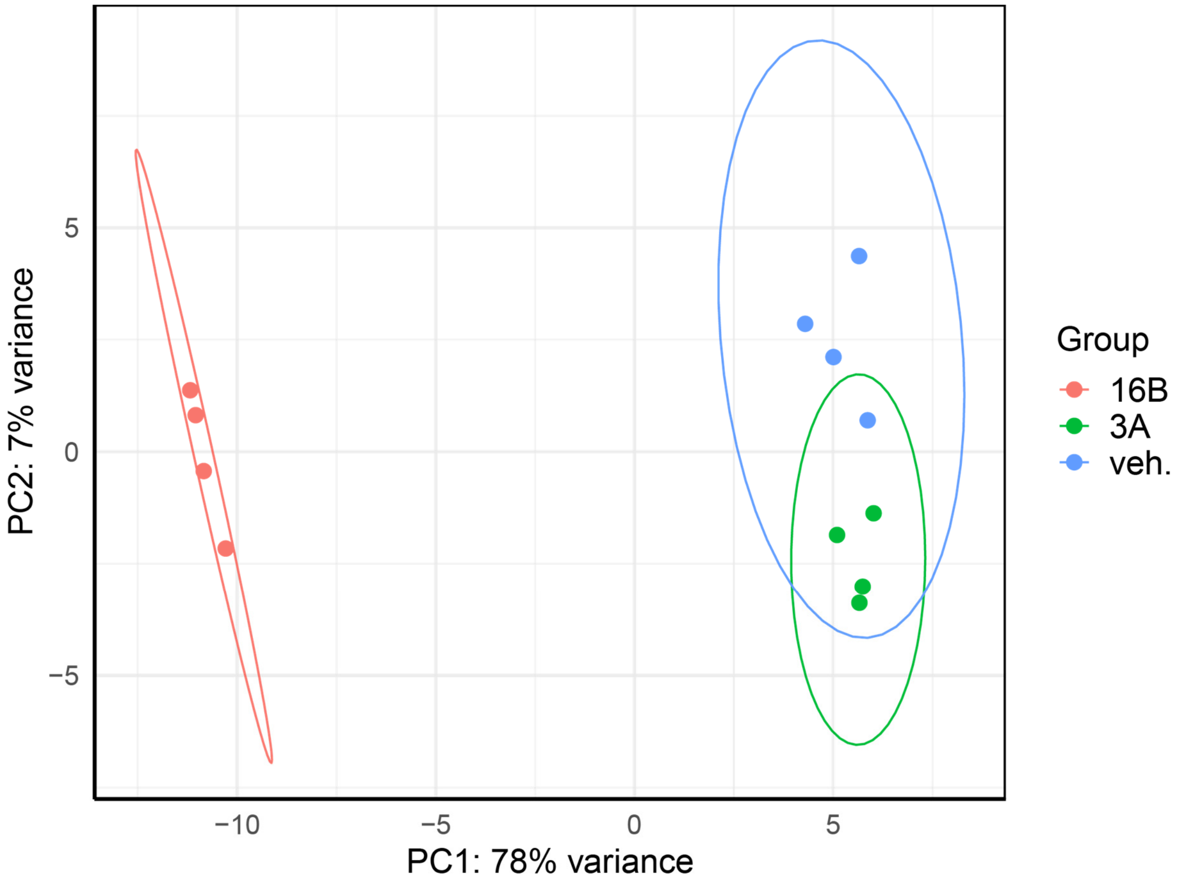

Supplementary Figure 8. PCA plot confirms clustering of samples according to treatment groups. Data underwent variance stabilizing transformation and was used to perform principal component analysis (PCA) using DESeq2 [55]. The resulting plot shows the samples in a 2D plane spanned by their first two principal components.

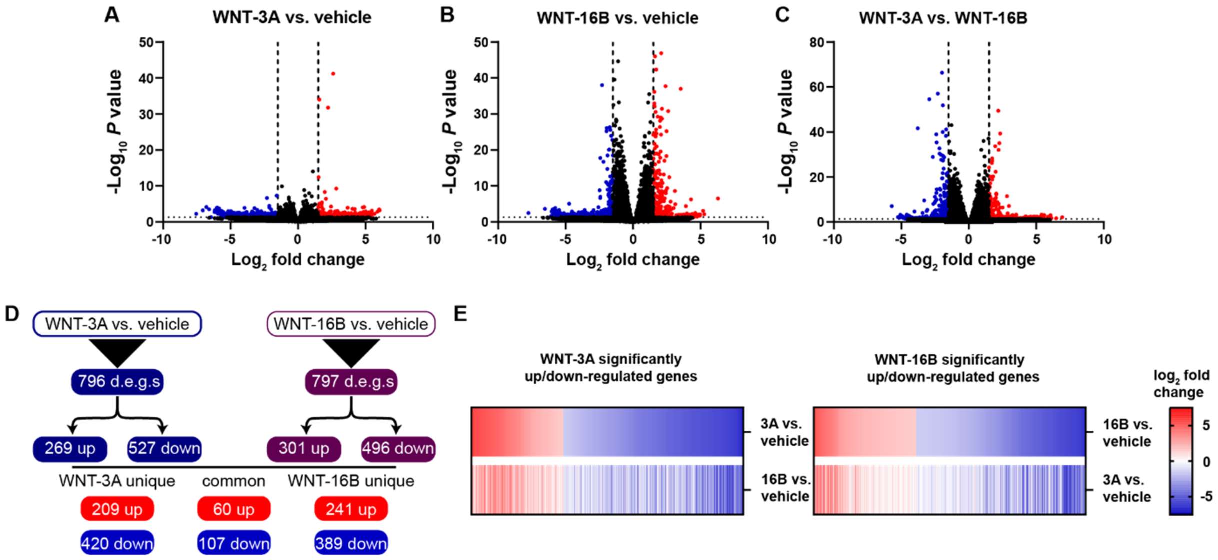

Supplementary Figure 9. Bulk mRNA sequencing reveals differential regulation of gene transcription by WNT-3A and WNT-16B in HEK293 cells. A-C. Volcano plots of differentially expressed genes (d.e.g.s) ($p < 0.05$; log2 fold change < -1.5 (blue)/ > 1.5 (red)) in HEK293 cells comparing WNT-3A treatment versus vehicle (A), WNT-16B versus vehicle (B), and WNT-3A versus WNT-16B (C). **D**. Descriptive statistics of differentially expressed genes identified by comparisons between samples treated with WNT-3A vs. vehicle and WNT-16B vs. vehicle. **E**. Heatmaps depicting the $\log_2$ fold expression change of all differentially regulated genes for WNT-3A vs. vehicle (left) or WNT-16B vs. vehicle (right) with the corresponding results for each respective gene from the other dataset (bottom row). All differentially expressed genes are listed in Supporting Data Table 1. Data summarize four independent experiments.

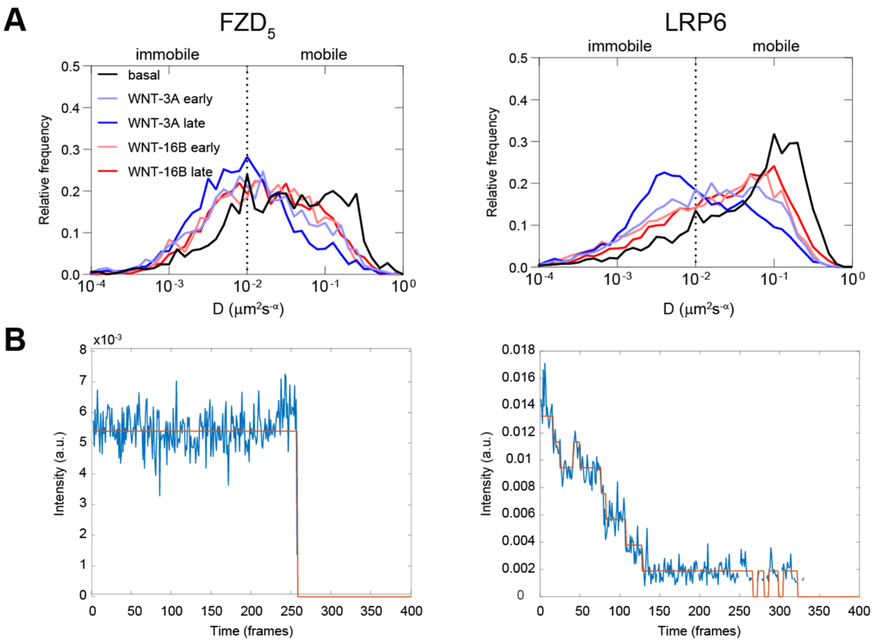

Supplementary Figure 10. Distribution of FZD_5 and LRP6 diffusion coefficients. A. Distributions of diffusion coefficient revealed by time-averaged mean squared displacement analysis at basal and following 100 nM WNT-3A and 100 nM WNT-16B stimulations. Early stimulation: 2-10 min, late stimulation: 11-25 min. n = 18, 25, 22, 17, 27 cells for FZD_5-LRP6 basal, WNT-3A early, WNT-3A late, WNT-16B early, and WNT-16B late, respectively, from six independent experiments. **B**. Representative examples of single-step (left) and multi-step photobleaching (right), indicating the presence of single and multiple molecules, respectively.

Supplementary table 1: Plasmids used which were not originally created in this study. Reference numbering refers to main text references.

HA-$FZD_{4/5/7}$-Nluc	Grätz et al. [38]<br><br>Grätz & Kowalski-Jahn et al. [14]
FLAG-DVL2	Gift from Vítězslav Bryja (Brno, CZ)
DVL1-HA	Gift from Vítězslav Bryja (Brno, CZ)
DVL3-HA	Gift from Vítězslav Bryja (Brno, CZ)
HA-FZD_5 $R^{6.32}$A-Nluc	Grätz & Kowalski-Jahn et al. [14]
HiBiT-$FZD_{4/5/7}$	Kozielewicz et al. [30]
8x SuperTOPFlash	Addgene #12456 [32]
pRL-TK (Renilla control plasmid)	Promega AF025846
β_2-Nluc	Bowin et al. [17]
ΔCRD-FZD_5-Nluc	Grätz et al. [38]
MESD	Gift from Stephane Angers (Toronto, CA)
HA-RNF43 (wt, R286W, D300G)	Gift from Vítězslav Bryja (Brno, CZ)
SNAP-β_2	Grimes et al. [51]
