# Supplementary material for: WNT-induced association of Frizzled and LRP6 is not sufficient for the initiation of WNT/β-catenin signaling
Source: Nat Commun. 2025 May 24;16:4848. doi: 10.1038/s41467-025-60096-7 (PMC12103576; doi:10.1038/s41467-025-60096-7)
Supplement: Supplementary file 2 — Description of Additional Supplementary Files [file 41467_2025_60096_MOESM2_ESM.pdf]

File Name: Supplementary Dataset 1.

Description: RNA sequencing data comparing datasets from vehicle-, WNT-3A- (300 ng/ml), and WNT-16B (300 ng/ml)-stimulated HEK293 cells with each other, including the baseMean, log2FoldChange, p-value, ENSEBL gene ID and gene name.

File Name: Supplementary Dataset 2.

Description: Gene ontology and KEGG pathway analyses for RNA sequencing datasets.

File Name: Supplementary Movie 1.

Description: Single-molecule fluorescence microscopy of SNAP-FZD_5 (magenta) and HALO-LRP6 (green) in CHO-K1 cells, untreated. Co-localization events appear as white. Playback in real time. Scale bar = 10 µm.

File Name: Supplementary Movie 2.

Description: Single-particle tracking of SNAP-FZD_5 (magenta) and HALO-LRP6 (green) in CHO-K1 cells, untreated, as derived from data shown in Supp. Movie 1. Each particle's traces are shown in the protein's respective color, co-locomotion is shown via blue traces. Playback in real time.

File Name: Supplementary Movie 3.

Description: Single-molecule fluorescence microscopy of SNAP-FZD_5 (magenta) and HALO-LRP6 (green) in CHO-K1 cells, early WNT-3A (100 nM) stimulation (within 10 min). Co-localization events appear as white. Playback in real time. Scale bar = 10 µm.

File Name: Supplementary Movie 4.

Description: Single-particle tracking of SNAP-FZD_5 (magenta) and HALO-LRP6 (green) in CHO-K1 cells, early WNT-3A (100 nM) stimulation (within 10 min), as derived from data shown in Supp. Movie 3. Each particle's traces are shown in the protein's respective color, co-locomotion is shown via blue traces. Playback in real time.

File Name: Supplementary Movie 5.

Description: Single-molecule fluorescence microscopy of SNAP-FZD_5 (magenta) and HALO-LRP6 (green) in CHO-K1 cells, late WNT-3A (100 nM) stimulation (within 10-30 min). Co-localization events appear as white. Playback in real time. Scale bar = 10 µm.

File Name: Supplementary Movie 6.

Description: Single-particle tracking of SNAP-FZD_5 (magenta) and HALO-LRP6 (green) in CHO-K1 cells, late WNT-3A (100 nM) stimulation (within 10-30 min), as derived from data shown in Supp. Movie 5.

Each particle's traces are shown in the protein's respective color, co-locomotion is shown via blue traces. Playback in real time.

File Name: Supplementary Movie 7.

Description: Single-molecule fluorescence microscopy of SNAP-FZD_5 (magenta) and HALO-LRP6 (green) in CHO-K1 cells, early WNT-16B (100 nM) stimulation (within 10 min). Co-localization events appear as white. Playback in real time. Scale bar = 10 µm.

File Name: Supplementary Movie 8.

Description: Single-particle tracking of SNAP-FZD_5 (magenta) and HALO-LRP6 (green) in CHO-K1 cells, early WNT-16B (100 nM) stimulation (within 10 min), as derived from data shown in Supp. Movie 7. Each particle's traces are shown in the protein's respective color, co-locomotion is shown via blue traces. Playback in real time.

File Name: Supplementary Movie 9.

Description: Single-molecule fluorescence microscopy of SNAP-FZD_5 (magenta) and HALO-LRP6 (green) in CHO-K1 cells, late WNT-16B (100 nM) stimulation (within 10-30 min). Co-localization events appear as white. Playback in real time. Scale bar = 10 µm.

File Name: Supplementary Movie 10.

Description: Single-particle tracking of SNAP-FZD_5 (magenta) and HALO-LRP6 (green) in CHO-K1 cells, late WNT-16B (100 nM) stimulation (within 10-30 min), as derived from data shown in Supp. Movie 9. Each particle's traces are shown in the protein's respective color, co-locomotion is shown via blue traces. Playback in real time.

File Name: Supplementary Movie 11.

Description: Single-molecule fluorescence microscopy of the β_2 adrenergic receptor (magenta) and HALO-LRP6 (green) in CHO-K1 cells, untreated. Co-localization events appear as white. Playback in real time. Scale bar = 10 µm.

File Name: Supplementary Movie 12.

Description: Single-particle tracking of the β_2 adrenergic receptor (magenta) and HALO-LRP6 (green) in CHO-K1 cells, untreated, as derived from data shown in Supp. Movie 11. Each particle's traces are shown in the protein's respective color, co-locomotion is shown via blue traces. Playback in real time.

File Name: Supplementary Movie 13.

Description: Single-molecule fluorescence microscopy of SNAP-FZD_5 (magenta) and HALO-LRP6-5A (green) in CHO-K1 cells, untreated. Co-localization events appear as white. Playback in real time. Scale bar = 10 µm.

File Name: Supplementary Movie 14.

Description: Single-particle tracking of SNAP-FZD_5 (magenta) and HALO-LRP6-5A (green) in CHO-K1 cells, untreated, as derived from data shown in Supp. Movie 13. Each particle's traces are shown in the protein's respective color, co-locomotion is shown via blue traces. Playback in real time.

File Name: Supplementary Movie 15.

Description: Single-molecule fluorescence microscopy of SNAP-FZD_5 (magenta) and HALO-LRP6-5A (green) in CHO-K1 cells, early WNT-3A (100 nM) stimulation (within 10 min). Co-localization events appear as white. Playback in real time. Scale bar = 10 µm.

File Name: Supplementary Movie 16.

Description: Single-particle tracking of SNAP-FZD_5 (magenta) and HALO-LRP6-5A (green) in CHO-K1 cells, early WNT-3A (100 nM) stimulation (within 10 min), as derived from data shown in Supp. Movie 15. Each particle's traces are shown in the protein's respective color, co-locomotion is shown via blue traces. Playback in real time.

File Name: Supplementary Movie 17.

Description: Single-molecule fluorescence microscopy of SNAP-FZD_5 (magenta) and HALO-LRP6-5A (green) in CHO-K1 cells, early WNT-3A (100 nM) stimulation (within 10-30 min). Co-localization events appear as white. Playback in real time. Scale bar = 10 µm.

File Name: Supplementary Movie 18.

Description: Single-particle tracking of SNAP-FZD_5 (magenta) and HALO-LRP6-5A (green) in CHO-K1 cells, early WNT-3A (100 nM) stimulation (within 10-30 min), as derived from data shown in Supp. Movie 17. Each particle's traces are shown in the protein's respective color, co-locomotion is shown via blue traces. Playback in real time.
