# Supplementary material for: WNT-induced association of Frizzled and LRP6 is not sufficient for the initiation of WNT/β-catenin signaling
Source: Nat Commun. 2025 May 24;16:4848. doi: 10.1038/s41467-025-60096-7 (PMC12103576; doi:10.1038/s41467-025-60096-7)
Supplement: Supplementary file 23 — Reporting Summary [file 41467_2025_60096_MOESM23_ESM.pdf]

nature portfolio

Corresponding author(s): Gunnar Schulte

Last updated by author(s): May 9, 2025

Reporting Summary

Nature Portfolio wishes to improve the reproducibility of the work that we publish. This form provides structure for consistency and transparency in reporting. For further information on Nature Portfolio policies, see our Editorial Policies and the Editorial Policy Checklist.

Statistics

For all statistical analyses, confirm that the following items are present in the figure legend, table legend, main text, or Methods section.

n/a	Confirmed	
☐	☒	The exact sample size (n) for each experimental group/condition, given as a discrete number and unit of measurement
☐	☒	A statement on whether measurements were taken from distinct samples or whether the same sample was measured repeatedly
☐	☒	The statistical test(s) used AND whether they are one- or two-sided<br>*Only common tests should be described solely by name; describe more complex techniques in the Methods section.*
☒	☐	A description of all covariates tested
☐	☒	A description of any assumptions or corrections, such as tests of normality and adjustment for multiple comparisons
☐	☒	A full description of the statistical parameters including central tendency (e.g. means) or other basic estimates (e.g. regression coefficient) AND variation (e.g. standard deviation) or associated estimates of uncertainty (e.g. confidence intervals)
☐	☒	For null hypothesis testing, the test statistic (e.g. F, t, r) with confidence intervals, effect sizes, degrees of freedom and P value noted<br>*Give P values as exact values whenever suitable.*
☒	☐	For Bayesian analysis, information on the choice of priors and Markov chain Monte Carlo settings
☒	☐	For hierarchical and complex designs, identification of the appropriate level for tests and full reporting of outcomes
☒	☐	Estimates of effect sizes (e.g. Cohen's d, Pearson's r), indicating how they were calculated

Our web collection on statistics for biologists contains articles on many of the points above.

Software and code

Policy information about availability of computer code

Data collection	Reader Control Software TECAN Spark plate reader, Image Lab for BioRad ChemiDoc, previosuly published R packages as indicated in Methods for RNA seq analysis, previously published Matlab code as indicated in the Methods section for analysis of single-particle tracking
Data analysis	MS Excel, Image Lab, GraphPad Prism 10.0, MatLab, R, FIJI

For manuscripts utilizing custom algorithms or software that are central to the research but not yet described in published literature, software must be made available to editors and reviewers. We strongly encourage code deposition in a community repository (e.g. GitHub). See the Nature Portfolio guidelines for submitting code & software for further information.

Data

Policy information about availability of data

All manuscripts must include a data availability statement. This statement should provide the following information, where applicable:

- Accession codes, unique identifiers, or web links for publicly available datasets
- A description of any restrictions on data availability
- For clinical datasets or third party data, please ensure that the statement adheres to our policy

Provide your data availability statement here.

Research involving human participants, their data, or biological material

Policy information about studies with human participants or human data. See also policy information about sex, gender (identity/presentation), and sexual orientation and race, ethnicity and racism.

Reporting on sex and gender	N/A
Reporting on race, ethnicity, or other socially relevant groupings	N/A
Population characteristics	N/A
Recruitment	N/A
Ethics oversight	N/A

Note that full information on the approval of the study protocol must also be provided in the manuscript.

Field-specific reporting

Please select the one below that is the best fit for your research. If you are not sure, read the appropriate sections before making your selection.

[x] Life sciences [ ] Behavioural & social sciences [ ] Ecological, evolutionary & environmental sciences

For a reference copy of the document with all sections, see nature.com/documents/nr-reporting-summary-flat.pdf

Life sciences study design

All studies must disclose on these points even when the disclosure is negative.

Sample size	No sample size calculations were performed. All experiments were replicated with at least three biological replicates (see figure legends)
Data exclusions	In general, no data were excluded. Single wells that were non-transfected were omitted from analysis when present.
Replication	Findings were well-reproducible across the biological replicates performed throughout the study.
Randomization	Randomization was not applicable to our study, as it focused solely on cell-based experiments investigating protein interactions. Such experiments do not inherently require randomization.
Blinding	Blinding was not relevant to cellular studies and blinding investigators during the procedures was technically not feasible.

Reporting for specific materials, systems and methods

We require information from authors about some types of materials, experimental systems and methods used in many studies. Here, indicate whether each material, system or method listed is relevant to your study. If you are not sure if a list item applies to your research, read the appropriate section before selecting a response.

Materials & experimental systems

n/a	Involved in the study	
[ ]	[x]	Antibodies
[ ]	[x]	Eukaryotic cell lines
[x]	[ ]	Palaeontology and archaeology
[x]	[ ]	Animals and other organisms
[x]	[ ]	Clinical data
[x]	[ ]	Dual use research of concern
[x]	[ ]	Plants

Methods

n/a	Involved in the study	
[x]	[ ]	ChIP-seq
[x]	[ ]	Flow cytometry
[x]	[ ]	MRI-based neuroimaging

Antibodies

Antibodies used	α-FLAG-M2, Sigma, F1804, or α-HA, abcam, ab9110, Lot No. GR3425636-2; anti-mouse-HRP, Thermo Fisher Scientific, 31430; anti-rabbit-HRP, Thermo Fisher Scientific, 31460; α-P-LRP6, Cell Signaling Tech., Cat. No. 2568, Lot No. 9; α-DVL2, Cell Signaling Tech., Cat. No. 3216, Lot No. 2; α-GAPDH, Cell Signaling Tech., Cat. No. 2118, Lot No. 61; α-P-β-catenin, Cell Signaling Tech., Cat. No. 9561, Lot No. 5; α-total β-catenin, BD Biosciences, Cat. No. 610154, Lot No. 3242871

Validation	Antibodies were validated by the suppliers as detailed on the product webpages.

Eukaryotic cell lines

Policy information about cell lines and Sex and Gender in Research

Cell line source(s)	HEK293A cells: ThermoFisher Scientific<br>ΔFZD1-10 and ΔLRP5/6 HEK293T cells were a kind gift from Benoit Vanhollebeke (PMID: 30026314)
Authentication	No
Mycoplasma contamination	Cell lines were regularly tested for mycoplasma contamination and found to be negative using the MycoStrip kit (InvivoGen)
Commonly misidentified lines (See ICLAC register)	None used.

Plants

Seed stocks	N/A
Novel plant genotypes	N/A
Authentication	N/A
